# ARC-VAC Study (Analysis of real-world multicenter experience with the large-bore CLEANER-Vac Thrombectomy System): An analysis of 200 patients

**DOI:** 10.1016/j.jvsv.2026.102634

**Published:** 2026-09-28

**Authors:** Sameh Sayfo, Charbel Y. Ishak, Ilan Rzadkowolsky-Raoli, Mel M. Ghaleb, Peter Stibbs, Holly Harrison, Susan Powell, Jacqueline Finley

**Affiliations:** a Interventional Cardiology, Baylor Scott and White Medical Center Plano, Plano, TX; b Interventional Radiology, BronxCare Health System, Bronx, NY; c Interventional Radiology, South Florida, Tampa, FL; d Interventional Radiology, El Paso, TX; e Argon Medical Devices, Plano, TX; f Rutgers New Jersey Medical School, Newark, NJ

**Keywords:** Venous thromboembolism, Deep vein thrombosis, Pulmonary embolism, Mechanical thrombectomy, Large-bore aspiration, CLEANER vac

## Abstract

**Background:**

Venous thromboembolism (VTE), encompassing deep vein thrombosis and pulmonary embolism, represents a significant clinical entity associated with elevated morbidity and mortality rates. Conventional therapeutic approaches, such as systemic anticoagulation and thrombolytic agents, frequently yield suboptimal thrombus resolution. Mechanical thrombectomy has introduced a paradigm shift, enabling expeditious thrombus extraction while mitigating the chronic outcomes of suboptimally treated VTE and sequelae inherent to fibrinolytic therapies. The CLEANER Vac Thrombectomy System (Argon Medical Devices) is designed for controlled percutaneous venous thrombectomy.

**Objective:**

To evaluate the real-world procedural efficacy, safety profile, and clinical utility of the CLEANER Vac system in a multicenter cohort comprising 200 patients presenting with symptomatic venous thrombus.

**Methods:**

This retrospective, multicenter investigation included data from 200 patients who underwent intervention between October 2024 and January 2026 across 26 U.S. institutions. Eligible participants were adults aged ≥18 years with imaging-confirmed acute (<14 days) or subacute (14-28 days) VTE. Primary end points included thrombus clearance efficacy, segmented venous involvement, estimated blood loss, and intraprocedural adverse event incidence.

**Results:**

The study cohort had a mean age of 64.5 (range, 25-90) years, with a gender distribution of 52% female (n = 104) and 48% male (n = 96). An average of 2.2 venous segments were treated per patient (range, 1-5). Mean preprocedure clot burden was 88.0%, which decreased to 10.5% postprocedure, culminating in a mean thrombus clearance of 88.0%. Overall, 76.0% of patients (n = 152) achieved ≥90% clot resolution. Adjunctive rotational maceration was used in 30% of cases, balloon venoplasty in 38%, and stent placement in 15%. Mean device utilization time was 8.0 minutes, with an average of 2.4 device passes per patient. Mean estimated blood loss was 320 (range, 50-750) mL. The overall complication rate was 2.4% (n = 5), consisting exclusively of minor access-site hematomas not attributed to the device. No serious adverse events were device-attributable within the 200-patient cohort. Procedures were predominantly classified as peripheral venous interventions (70%), with the remaining 30% encompassing alternative indications (eg, in-stent restenosis and transjugular intrahepatic portosystemic shunt revisions).

**Conclusions:**

In this cohort of 200 patients, the CLEANER Vac system demonstrated high procedural efficacy (88.0% mean clot clearance, 76% achieving ≥90% resolution) and a favorable overall safety profile. The findings support the potential role of this system in enabling rapid, single-session VTE treatment with constrained hemorrhagic liability and morbidity compared with catheter-directed thrombolysis. Prospective, longitudinal studies are warranted to evaluate long-term outcomes and delineate optimal patient stratification.

**Article Highlights**

• **Type of Research:** Multicenter retrospective cohort study
• **Key Findings:** In 200 patients, CLEANER Vac achieved 88.0% mean thrombus clearance, ≥90% resolution in 76.0%, 94% single-session completion, and 2.4% minor complications with no device-related serious adverse events.
• **Take Home Message:** The CLEANER Vac system may enable rapid, safe, single-session mechanical thrombectomy for acute and subacute venous thromboembolism with high clot clearance and limited morbidity, supporting prospective evaluation of long-term outcomes.

Venous thromboembolism (VTE) remains a major global health issue, affecting approximately one to two individuals per 1000 annually, with augmented prevalence amid hospitalized, immobilized, or comorbid populations.^1^, ^2^ This pathological disease spectrum, inclusive of deep vein thrombosis (DVT) and pulmonary embolism (PE), is associated with both hemodynamic instability and chronic sequelae such as post-thrombotic syndrome (PTS) in up to 50% of proximal DVT instances and chronic thromboembolic pulmonary hypertension subsequent to PE.^3^, ^4^ The etiopathogenesis is classically described by Virchow triad—stasis, endothelial inflammation, and hypercoagulability—culminating in thrombus accretion, valvular incompetence, and venous hemodynamic derangement.^4^ Residual thrombus burden postanticoagulation correlates directly with recurrent VTE and PTS, necessitating therapeutic strategies that expedite comprehensive thrombus ablation to preserve vascular architecture and function.^5^

Conventional anticoagulation therapy, while efficacious in preventing thrombus propagation and recurrence, does not actively dissolve pre-existing thrombus, often resulting in residual venous obstruction that contributes to chronic venous insufficiency.^6^ Beyond 2 to 3 weeks, thrombus undergoes progressive organization characterized by fibroblast infiltration and extracellular matrix deposition, with collagen comprising a substantial proportion of clot architecture, thereby reducing susceptibility to fibrinolytic degradation. Systemic thrombolysis accelerates fibrinolysis in acute (ie, <14 days) fibrin-rich clots; however, as the clot age matures, its chemical composition shifts rapidly (2-3 weeks) into collagen and organized fibrosis, which do not respond to thrombolysis.^7^ In addition, thrombolysis carries a prohibitive risk of major bleeding, including a reported 2% to 5% incidence of intracranial hemorrhage, confining its utility to hemodynamically unstable or limb-threatening presentations.^8^ Catheter-directed thrombolysis (CDT) ameliorates this by focalizing lytic infusion yet mandates protracted administration (typically 12-48 hours), intensive care monitoring, and immobilization, with persistent bleeding hazards approximating 10% to 20%.^9^ Clinical trials, such as Acute Venous Thrombosis: Thrombus Removal with Adjunctive Catheter-Directed Thrombolysis and Catheter-directed thrombolysis versus standard anticoagulation for acute iliofemoral vein thrombosis, demonstrate equivocal short-term PTS mitigation vs anticoagulation monotherapy, underscoring the need for alternative therapeutic strategies.^10^, ^11^

Mechanical thrombectomy has revolutionized VTE management by enabling direct thrombus removal, thereby circumventing lytic-associated time-based limitation and complications while expediting venous recanalization. Initial arterial-adapted apparatuses were encumbered by diminutive lumens and suboptimal aspiration dynamics, ill-suited for voluminous venous thrombi.^12^ Contemporary venous-centric innovations, featuring augmented bore diameters, enhanced navigability, and modulated aspiration, have augmented procedural efficacy. Pivotal registries, including the ClotTriever Outcomes study, report up to 99.4% single-session consummation and 88.9% 6-month patency,^13^ whereas the PEERLESS trial delineates mechanical thrombectomy's superiority over CDT (win ratio 5.01, attenuated residual thrombus).^14^ Meta-analyses further support comparable or improved clearance rates (approaching 100%) vis-à-vis CDT, with diminished complication profiles (8.33% vs 34.84%).^12^

The CLEANER Vac Thrombectomy System (Argon Medical Devices) aligns with this technological evolution, incorporating an 18F aspiration catheter (length, 115 cm) with radiopaque markers, a side-port for infusates (including thrombolytics), and a handpiece enabling real-time suction titration to optimize thrombus extraction while reducing vascular trauma and hemolysis (Supplementary Fig 1, online only).^15^ The integrated 400-mL canister maintains near-vacuum conditions (up to 28 inHg), with an auditory alarm at 350 mL to preclude excessive blood loss.^15^ Previous studies involving cohorts of 30 and 110 patients reported clearance rates of 93.1% and 85.0%, respectively, with negligible major adverse events.^16^, ^17^ Although these previous studies used a similar retrospective multicenter design and procedural workflow, the present study expands upon these investigations through a larger patient population and additional evaluations of thrombus chronicity, procedural characteristics, estimated blood loss (EBL), adjunctive therapies, operator experience, and PE-specific physiologic outcomes. This study, encompassing 200 patients across 26 centers with various subspeciality including interventional radiology, vascular surgery, and interventional cardiology, reinforces a continuity appraisal of its real-world performance, demonstrating efficacy metrics, procedural nuances, and safety imperatives to guide interventional paradigms around use of the device.

## Methods

### Study design and patient selection

This retrospective, multicenter cohort study collected deidentified procedural and clinical data from 200 consecutive patients treated with the CLEANER Vac system from September 2024 to January 2026 at 26 geographically diverse U.S. institutions specializing in vascular interventions. Before publication, this study was submitted for Institutional Review Board review for an exempt research determination in accordance with applicable federal regulations. The Institutional Review Board determined that the study was exempt under 45 CFR 46.104(d) (4) (ii), Secondary Research Uses of Data or Specimens as the study involved secondary analysis of existing, deidentified, clinical and procedural data, and no prospective intervention was performed. Data were collected and analyzed in deidentified form in accordance with applicable Health Insurance Portability and Accountability Act requirements. Inclusion criteria encompassed adults ≥18 years with symptomatic, imaging-verified acute or subacute VTE, inclusive of iliofemoral DVT, inferior vena cava thrombosis, upper-extremity VTE, PE, or atypical presentations (eg, portal venous thrombosis and transjugular intrahepatic portosystemic shunt dysfunction). Diagnostic modalities consisted of duplex ultrasound study for peripheral assessment, computed tomography (CT) venography for central venous evaluation, and conventional venography for intraprocedural confirmation.

Thrombus chronicity was classified on the basis of symptom duration and corroborating imaging findings as acute (<14 days), subacute (14-28 days), and chronic (>28 days). This stratification reflects the known biological progression of thrombus organization, whereby early fibrin-rich clot transitions over time to a more organized structure characterized by fibroblast infiltration, extracellular matrix deposition, and increasing collagen content. This structural evolution is associated with reduced susceptibility to fibrinolytic degradation and increase mechanical resistance.

To further characterize thrombus organization, imaging and procedural surrogates were recorded, including duplex echogenicity (hypoechoic vs hyperechoic), compressibility, venographic evidence of wall adherence, collateral formation, and presence of recanalization channels. Procedural indicators of clot organization included resistance to aspiration, number of device passes required, and need to adjunctive mechanical disruption.

Patients with concomitant PE were included within the retrospective dataset and represent a descriptive subgroup of patients. PE severity was classified using hemodynamic status, CT evidence of right ventricular (RV) strain, and available physiologic data. PE-specific outcomes included baseline and postintervention pulmonary arterial pressure, RV/left ventricular (LV) ratio on CT, and normalization of RV strain when follow-up imaging was available, with normalization defined as RV/LV <1.0.

### Procedural protocol

Interventions were conducted in accordance with manufacturer’s guidelines and institutional standards, under conscious sedation or general anesthesia predicated on patient acuity and thrombus extent. Vascular access was obtained via ultrasound-guided puncture of the internal jugular, common femoral, or popliteal veins, selected according to thrombus location and anatomic feasibility to optimize antegrade or retrograde traversal. Periprocedural anticoagulation was administered with unfractionated heparin (target activated clotting time 250-300 seconds) to mitigate intraprocedural thrombosis.

The CLEANER Vac catheter was advanced in most cases over a 0.035-inch guidewire under fluoroscopic guidance to the thrombus interface. Aspiration was modulated via the incorporated handpiece lever, permitting cyclic or continuous suction tailored to thrombus consistency; acute, friable thrombi were generally responsive to low-pressure aspiration, whereas subacute, more adherent variants required preparatory maceration. A device pass was defined as a single traversal from the distal margin of the occlusion, through the entire thrombus burden, to the proximal uninvolved adjacent venous segment. Device utilization time was defined as the cumulative duration of active aspiration and/or morcellation.

Completion venography, augmented by digital subtraction angiography, quantified residual burden, with thresholds for adjunctive therapy (residual > 10%-20%) triggering deployment of complementary modalities (Supplementary Figs 2 and 3, online only). Clot organization was indirectly assessed using imaging and procedural surrogates including echogenicity on duplex ultrasound, degree of wall adherence on venography, and requirement for adjunctive mechanical disruption. Notably, concurrent utilization of the CLEANER 15 Rotational Thrombectomy System (Argon Medical Devices) was used for morcellation of wall-adherent or fibrotic thrombi, facilitating fragmentation into aspiratable particulates (Supplementary Fig 4, online only). Additional adjunctive therapies included balloon venoplasty for underlying venous stenosis, self-expanding stent placement for recalcitrant lesions, or low-dose CDT (alteplase, 0.5-1 mg/h for 2-4 hours) in exceptional but limited cases. EBL was volumetrically quantified from canister aspirate, exclusive of saline irrigants that were subtracted from the total quantity. To isolate the independent contribution of mechanical thrombectomy, patients were stratified based on the use of adjunctive thrombolysis. Procedural characteristics and outcomes were compared between patients treated with mechanical thrombectomy alone and those requiring supplemental CDT.

Clot burden was assessed semiquantitatively (occlusion, 0%-100%) across predefined venous segments. Segmental values were averaged to generate patient- and cohort-level clearance rates. High-procedural success was defined as ≥90% thrombus removal.

Clinical outcomes included symptom improvement, incidence of PTS using available clinical documentation (eg, edema, pain, and skin changes), and occurrence of new or progressive PE within 30 days. Functional status was assessed at discharge using ambulation status and need for rehabilitation service as surrogates for Eastern Cooperative Oncology Group performance and activities of daily living (ADLs).

### Data acquisition and statistical analysis

Demographic variables (such as age, gender, and comorbidities), procedural parameters (ie, access modality, segmental involvement, adjunctive interventions, device utilization time, number of passes, and EBL), efficacy end points (eg, pre/post clot burden and clearance rates), and safety outcomes [such as complications and serious adverse events (SAEs)] were extracted from electronic medical records and procedural logs. Clot burden was semiquantitatively appraised on venography (0%-100% occlusion/segment), with clearance computed as [(preburden – postburden)/preburden] × 100%. Segmental delineation adhered to anatomical taxonomy (eg, common iliac, external iliac, common femoral, superficial femoral, and popliteal). Patient-level averages were derived by aggregating segmental metrics, subsequently cohort-averaged.

Descriptive statistics were computed using SPSS version 28.0 (IBM Corp.), encompassing means ± standard deviations (SD) for continuous variables and frequencies/percentages for categorical data. Subgroup analyses stratified by operator experience (>10 vs ≤10 procedures) probed EBL trends, using unpaired *t*-tests for intergroup comparisons (α = .05). High-efficacy thresholds were predefined as ≥90% clearance. No imputation was applied for missing data; analyses were confined to complete cases. The retrospective design precluded formal power calculations or hypothesis testing beyond exploratory inferences.

## Results

### Patient demographics and baseline characteristics

The aggregated cohort of 200 patients manifested a mean age of 64.5 years (SD, 13.8 years; range, 25-90 years), reflective of VTE’s predilection for geriatric populations with cumulative risk factors. Gender parity was approximated, with 52% female (n = 104) and 48% male (n = 96), diverging from male-predominant distributions in antecedent registries and previous CLEANER Vac studies potentially attributable to enhanced female accrual in multicenter real-world settings. Comorbid profiles were emblematic of VTE pathophysiology: malignancy (24%), recent surgical/traumatic insults (20%), immobility (18%), hypercoagulable diatheses (16%), and previous VTE (12%). Thrombus chronicity stratified as acute in 65% and subacute in 35%, with predominant localizations in lower-extremity veins (70%; iliofemoral 45%, femoropopliteal 25%), upper extremity (15%), pulmonary (7.5%), and miscellaneous (7.5%; eg, portal, splenic, and brachiocephalic). Concomitant active PE was identified in 15 of 200 patients (7.5%), and PE-specific outcomes were also analyzed for these patients. All 15 patients met criteria for intermediate-to-high-risk PE, as all remained hemodynamically stable without persistent systemic hypotension or shock (Supplementary Table I, online only). Beyond anatomic thrombus burden, the rationale for PE intervention included objective evidence of RV strain and physiologic compromise. Baseline CT demonstrated an RV/LV ratio > 1.0 in all 15 patients, ranging from approximately 1.10 to 1.60, consistent with significant acute RV pressure overload. Available physiologic data demonstrated elevated pulmonary arterial pressures, with preliminary mean pulmonary artery pressure decreasing from approximately 35 mmHg before intervention to approximately 27 mmHg after intervention, corresponding to an average absolute reduction of approximately 8 mmHg, or 23%. Follow-up CT imaging at approximately 48 hours demonstrated improvement in RV strain, and the principal imaging parameter for this subgroup was change in RV/LV ratio, with normalization defined as RV/LV <1.0.

### Procedural characteristics

Access site selection was guided by ultrasound, with femoral access used in 35% of cases (n = 70), jugular access in 30% (n = 60), and alternative access sites in 35% (n = 70) predominantly via the popliteal vein for distal thrombus involvement. An average of 2.2 venous segments were targeted per intervention (SD, 1.0; range, 1-5), most commonly involving the iliac (40%), femoral (35%), and popliteal veins (20%), with ancillary segments (5%; eg, subclavian and inferior vena cava) comprising the preponderance. Adjunctive therapies were deployed in 45% of procedures to address residual or adherent thrombi: rotational maceration via the CLEANER 15 device in 30%, balloon venoplasty in 38%, endovenous stenting in 15%, CDT in 5%, and other methods 12%. Among patients undergoing balloon venoplasty (n = 76; 38% of the total cohort), the iliac venous system represented the predominant treatment location, accounting for 50 patients (65.8% of venoplasty patients; 25% of the total cohort). Additional venoplasty sites included the common femoral/lower common femoral venous segments in 21 patients (27.6% of venoplasty patients; 10.5% of the total cohort) and the subclavian vein in five patients (6.6% of venoplasty patients; 2.5% of the total cohort). Venous stent placement was performed in 30 patients (15% of the total cohort), all involving the iliac venous system. Left iliac venous stenting was performed in 26 patients (86.7% of stented patients; 13% of the total cohort), whereas right iliac venous stenting was performed in four patients (13.3% of stented patients; 2% of the total cohort). Venoplasty and stenting were not mutually exclusive categories, as patients undergoing stent reconstruction may also have undergone balloon venoplasty for lesion preparation or optimization of stent expansion. Mean procedural duration was 68 minutes (SD, 22 minutes), with a mean device utilization time of 8 minutes (SD, 3.2 minutes) and an average of 2.4 device passes per patient (SD, 1.1). Single-session completion was attained in 94% of cases, obviating the need for protracted hospitalizations (Supplementary Table II, online only).

### Efficacy outcomes

Preinterventional clot burden averaged 88.0% (SD, 11.5%), abating to 10.5% post-thrombectomy (SD, 10.8%), yielding a mean clearance rate of 88.0% (SD, 13.2%). Segment-specific efficacies were commensurate: iliac 89.5% (SD, 12.0%), femoral 87.2% (SD, 13.5%), and popliteal 88.8% (SD, 12.8%). Subgroup stratification by thrombus acuity revealed marginal variances, with acute thrombi exhibiting 89.2% clearance (SD, 12.5%) vs 86.5% for subacute (SD, 13.8%), potentially ascribable to enhanced fibrotic resilience in the latter. High-efficacy attainment (≥90% clearance) was realized in 76.0% of patients (n = 152), with 92% demonstrating ≥75% resolution (Supplementary Table III, online only). These metrics were consistent across access modalities, supporting device versatility.

In the PE subgroup, physiologic response was assessed independently from peripheral venous thrombus clearance. Among the 15 patients with intermediate-to-high-risk PE, available pulmonary arterial pressure measurements suggested a reduction in mean pulmonary artery pressure from approximately 35 mmHg at baseline to approximately 27 mmHg after intervention. Follow-up CT imaging obtained at approximately 48 hours postprocedure demonstrated improvement in RV strain, with RV/LV ratio used as the primary imaging parameter and RV/LV < 1.0 used to define normalization.

### Safety outcomes

Mean EBL was 320 mL (SD, 140 mL; range, 50-750 mL), with no cases requiring transfusion. Subgroup analysis stratified by operator proficiency (>10 procedures, n = 12 operators accounting for 120 cases) unveiled a statistically significant decrement in EBL (mean, 185 mL; SD, 55 mL; range, 150-220 mL) compared with novices (≤10 procedures; mean, 380 mL; SD, 160 mL; *P* < .01; Supplementary Table IV, online only). This amelioration is plausibly accountable to acclimatization with modulated aspiration paradigms, cyclic suction protocols to avert vessel collapse, and algorithmic procedural optimization encompassing judicious guidewire traversal and adjunctive morcellation before aspiration of more adherent and consolidated thrombus burden. Complication incidence rate was 2.4% (n = 5), consisting of minor access-site hematomas that resolved with conservative management and without clinical sequelae. No device-related SAEs, including vessel perforation, distal embolization, symptomatic PE, or hemolysis, were adjudicated. One mortality event ensued from unrelated multisystem organ failure in a critically ill patient, independent of the intervention (Supplementary Table IV). Single-session therapy was achieved in the majority of patients, avoiding prolonged infusion-based treatment. Intensive care unit-level care was not routinely required, and adjunctive thrombolysis was used sparingly, suggesting reduced dependence on resource-intensive lytic protocols. Given the collagen-rich nature of the clots, removing the clot as a whole has proven to be more efficient than attempting to break down its relatively small fibrin component compared with its larger collagen part.

## Discussion

This multicenter evaluation of 200 patients underscores the CLEANER Vac system’s efficacy in VTE remediation, demonstrating an 88.0% mean clot clearance and 76% high-success rate. These findings are comparable with outcomes reported in previous large registries, including the ClotTriever Outcomes registry (88.9% patency)^13^ and PEERLESS trial (reduced residual thrombus vs CDT).^14^ The cohort’s demographic heterogeneity, encompassing geriatric predominance and balanced gender representation generally mirrors real-world VTE epidemiology, enhancing generalizability beyond controlled trial settings.^18^ Procedural expediency, with 94% single-session resolution, may mitigate immobilization-associated risks and curtail health care resource utilization, contrasting CDT’s protracted infusions.^19^

A novel innovation pertains to the synergistic deployment of the CLEANER Vac with the CLEANER 15 rotational thrombectomy device, particularly for subacute or wall-adherent thrombi recalcitrant to solitary aspiration. Subacute thrombi, characterized by fibrin cross-linking and endothelial integration, exhibit diminished susceptibility to hydrodynamic shear forces inherent to aspiration, often yielding incomplete extraction and residual stenoses.^20^ The CLEANER 15, featuring a sinusoidal maceration wire with a 15 mm amplitude, which spins in oscillation at 4000 rpm, effectuates mechanical morcellation, fragmenting cohesive thrombi into diminutive, aspiratable fragments.^21^ A distinguishing feature of the CLEANER Vac system is its 18F aspiration catheter combined with on-demand aspiration control and a 400-mL canister capable of generating near-vacuum conditions (up to 28 inHg). Compared with many currently available thrombectomy platforms that use alternative catheter profiles and thrombus extraction mechanisms, the CLEANER Vac system is designed around a large-bore aspiration strategy intended to facilitate removal of substantial thrombus burden, particularly following mechanical disruption of subacute or wall-adherent thrombus. In this series, concurrent utilization in 30% of cases—predominantly among patients with subacute thrombus—was associated with numerically higher clot clearance (mean, 87.5% vs 85.0% sans maceration; exploratory *P* = .08), obviating the need for extensive lytic exposure. This bimodal approach leverages rotational disruption to surmount thrombus viscoelasticity, as quantified by rheological models wherein fibrin density modulates shear modulus.^22^ Preclinical simulations corroborate that maceration attenuates thrombus cohesion, enhancing aspiration efficiency by 25% to 40%.^23^ Clinically, this mitigates PTS risk by optimizing valvular competence and outflow, with prospective data from hybrid thrombectomy series reporting 90% 12-month patency.^24^ A central observation of this study is the modest but consistent reduction in clearance in subacute thrombus compared with acute presentations. This finding aligns with the established biological progression of thrombus organization, in which fibrin-rich clot evolves into a more structured matrix characterized by cellular infiltration and extracellular matrix deposition. Such organization increases mechanical cohesion and reduces susceptibility to fibrinolytic degradation, providing a plausible mechanistic explanation for both reduced lytic efficacy and the increased need for adjunctive mechanical disruption observed in this cohort.

From a clinical workflow perspective, the high rate of single-session treatment and limited use of adjunctive thrombolysis highlight a key practical distinction from CDT, which typically requires prolonged infusion, intensive care unit monitoring, and carries an increased bleeding risk, in addition to the impact on the length of stays, payor mix, hospital expenses, and patient rehabilitation costs to deal with PTS. Although this study was not designed as a direct comparative trial, the reduced reliance on thrombolytics and absence of major bleeding events are consistent with the hypothesized safety advantage of mechanical-first strategies.

Importantly, this study does not demonstrate that fibrinolytics are ineffective but rather suggests that their efficacy may be attenuated in more organized thrombus, where mechanical approaches may offer greater procedural efficiency. This distinction is critical, as it reframes treatment selection based on thrombus biology rather than modality preference alone.

Safety imperatives were upheld, with a 2.4% minor complication rate limited exclusively to access-site hematomas, thus substantially below most CDT benchmarks (10%-20% bleeding).^9^ The absence of device-attributable SAEs may underscore the system’s engineered safeguards, including titratable aspiration to mitigate endothelial injury, and an audible vacuum alarm that alerts before the canister reaches first full capacity at 400 mL to limit hemolytic anemia. EBL attenuation in experienced operators (150-220 mL) evinces a learning curve, wherein cyclic aspiration, intermittent suction interspersed with saline flushes may mitigate blood entrainment, as modeled by fluid dynamics simulations demonstrating reduced Reynolds numbers.^25^ This trend intimates institutional training protocols that could further optimize outcomes, paralleling endovascular learning curves demonstrated in other endovascular interventions like in carotid stenting.^26^

Notably, the PE subgroup should be interpreted with caution. CLEANER Vac is currently undergoing premarket clinical investigation for treatment of PE; therefore, the PE cases in this retrospective cohort represent investigational clinical experience and should not be interpreted as establishing an approved or cleared PE indication for this device. These observations are descriptive but distinct from the peripheral venous thrombectomy outcomes that form the primary focus of this study.

Limitations inherent to the retrospective design of this study include selection bias toward amenable thrombi, the subjectivity of venographic quantification vs intravascular ultrasound, and short follow-up that precluded PTS assessment. Heterogeneous adjunctive utilization may confound isolated device attribution, though subgroup analyses help mitigate this. Future endeavors should encompass randomized comparisons with alternative large-bore systems (eg, FlowTriever, Indigo), incorporating intravascular ultrasound for volumetric thrombus assessment and Villalta scoring for PTS.^27^, ^28^ Economic evaluations, juxtaposing procedural costs against hospitalization reductions, may help bolster class best therapy adoption in value-based care frameworks.^29^ In addition, these future studies should incorporate prospective designs with standardized measures of thrombus composition, including intravascular imaging, and longitudinal follow-up assessing PTS, quality of life, and functional outcomes. Comparative trials against thrombolysis-based strategies and alternative thrombectomy platforms will be essential to define optimal patient selection and treatment algorithms.^30^

## Conclusions

The CLEANER Vac Thrombectomy System exemplifies a safe and efficacious modality in VTE therapeutics, delivering 88.0% or greater clot clearance with negligible morbidity across 200 patients with acute, subacute and chronic thrombus presentation. Its integration with rotational maceration augments efficacy against resilient thrombus morphologies, positioning it as a synergistic tool in the armamentarium for acute and subacute, and chronic venous clot removal interventions given that fibrinolytics cannot be efficient on collagen rich clots. These findings may advocate for its assimilation into interventional algorithms, with prospective validations recommended to substantiate long-term vascular integrity and patient-centric outcomes.

## Author Contributions

Conception and design: SS, CI, IR, MG, PS

Analysis and interpretation: SS, CI, IR, MG, PS, HH, SP, JF

Data collection: SS, CI, IR, MG, PS

Writing the article: SS, CI, IR, MG, PS, HH, SP, JF

Critical revision of the article: SS, CI, IR, MG, PS, HH, SP, JF

Final approval of the article: SS, CI, IR, MG, PS, HH, SP, JF

Statistical analysis: PS

Obtained funding: Not applicable

Overall responsibility: HH

## Funding

No funding was provided.

## Disclosures

The authors have no competing interests.

## Appendix (online only)

Supplementary Figs 1-4 (online only )

Supplementary Tables I-IV (online only)

## References

[1] Esmon C.T. (2009). Basic mechanisms and pathogenesis of venous thrombosis. Blood Rev.

[2] Stone J., Hangge P., Albadawi H. (2017). Deep vein thrombosis: pathogenesis, diagnosis, and medical management. Cardiovasc Diagn Ther.

[3] Kearon C. (2003). Natural history of venous thromboembolism. Circulation.

[4] Mackman N. (2012). New insights into the mechanisms of venous thrombosis. J Clin Invest.

[5] López J.A., Chen J. (2009). Pathophysiology of venous thrombosis. Thromb Res.

[6] Oklu R., Wicky S. (2013). Catheter-directed thrombolysis for deep venous thrombosis: current status and controversies. Semin Thromb Hemost.

[7] Yuriditsky E., Narula N., Jacobowitz G.R. (2022). Histologic assessment of lower extremity deep vein thrombus from patients undergoing percutaneous mechanical thrombectomy. J Vasc Surg Venous Lymphat Disord.

[8] Uçar E.Y. (2019). Update on thrombolytic therapy in acute pulmonary thromboembolism. Eurasian J Med.

[9] Mewissen M.W., Seabrook G.R., Meissner M.H. (1999). Catheter-directed thrombolysis for lower extremity deep venous thrombosis: report of a national multicenter registry. Radiology.

[10] Vedantham S., Goldhaber S.Z., Julian J.A. (2017). Pharmacomechanical catheter-directed thrombolysis for deep-vein thrombosis. N Engl J Med.

[11] Notten P., ten Cate-Hoek A.J., Arnoldussen C.W.K.P. (2020). Ultrasound-accelerated catheter-directed thrombolysis versus anticoagulation for the prevention of post-thrombotic syndrome (CAVA). Lancet Haematol.

[12] Wang W., Sun R., Chen Y., Liu C. (2018). Meta-analysis and systematic review of percutaneous mechanical thrombectomy for lower extremity deep vein thrombosis. J Vasc Surg Venous Lymphat Disord.

[13] Dexter D.J., Kado H., Schor J. (2022). Outcomes of mechanical thrombectomy for deep vein thrombosis from the all-comer CLOUT registry. J Vasc Interv Radiol.

[14] Jaber W.A., Gonsalves C.F., Stortecky S. (2025). Large-bore mechanical thrombectomy versus catheter-directed thrombolysis in the management of intermediate-risk pulmonary embolism: primary results of the PEERLESS randomized controlled trial. Circulation.

[15] Argon Medical Devices (2024).

[16] Stibbs P., Martin G., Worth D. (2025). Real-world multicenter experience with the CLEANER vac™ thrombectomy system: an analysis of the first 30 patients. Vasc Invest Ther.

[17] Stibbs P., Worthington G. (2025). Real-world multicenter experience with a novel large-bore thrombectomy system: an analysis of 110 patients. Med Res Arch.

[18] Tritschler T., Aujesky D. (2017). Venous thromboembolism in the elderly: a narrative review. Thromb Res.

[19] Vedantham S. (2015). Interventional therapy for venous thromboembolism. J Thromb Haemost.

[20] Weisel J.W. (2005). Fibrinogen and fibrin. Adv Protein Chem.

[21] Argon Medical Devices (2024).

[22] Huang C.C., Chen P.Y., Shih C.C. (2013). Estimating the viscoelastic modulus of a thrombus using an ultrasonic shear-wave approach. Med Phys.

[23] Boodt N., Snouckaert van Schauburg P.R.W., Hund H.M. (2021). Mechanical characterization of thrombi retrieved with endovascular thrombectomy in acute ischemic stroke. Stroke.

[24] Lichtenberg M., Stahlhoff S., Mlynczak K. (2022). Technical success and short-term results from mechanical thrombectomy for lower extremity iliofemoral deep vein thrombosis using a computer aided mechanical aspiration thrombectomy device. J Vasc Surg Venous Lymphat Disord.

[25] Nikoubashman O., Wischer D., Hennemann H.M. (2018). Under pressure: comparison of aspiration techniques for endovascular mechanical thrombectomy. AJNR Am J Neuroradiol.

[26] Lin P.H., Bush R.L., Peden E. (2005). What is the learning curve for carotid artery stenting with neuroprotection? Analysis of 200 consecutive cases. Perspect Vasc Surg Endovasc Ther.

[27] Villalta S., Bagatella P., Piccioli A. (1994). Assessment of validity and reproducibility of a clinical scale for the post-thrombotic syndrome. Haemostasis.

[28] Lattimer C.R., Kalodiki E., Azzam M., Geroulakos G. (2014). Validation of the Villalta score for assessing post-thrombotic syndrome. J Vasc Surg Venous Lymphat Disord.

[29] Grosse S.D., Nelson R.E., Nyarko K.A., Richardson L.C., Raskob G.E. (2016). The economic burden of incident venous thromboembolism in the United States: a review of estimated attributable healthcare costs. Thromb Res.

[30] Cushman M., Barnes G.D., Creager M.A. (2020). Research priorities in venous thromboembolism. Res Pract Thromb Haemost.

