# Supplementary material for: ARC-VAC Study (Analysis of real-world multicenter experience with the large-bore CLEANER-Vac Thrombectomy System): An analysis of 200 patients
Source: J Vasc Surg Venous Lymphat Disord. 2026 Sep 28;15(1):102634. doi: 10.1016/j.jvsv.2026.102634 (PMC13720463; doi:10.1016/j.jvsv.2026.102634)
Supplement: Supplementary file 1 [file mmc1.docx]

**Supplemental Figure 1: CLEANER Vac™ Thrombectomy System (Argon Medical Devices)**

Cleaner Vac Thrombectomy System| Argon Medical

Supplemental Figure 1: The CLEANER Vac™ Thrombectomy System, featuring an 18-French large-bore aspiration catheter with integrated handpiece for controlled suction, used as the primary device in the 200-patient multicenter study. (Image courtesy of Argon Medical Devices.)

**Supplemental Figure 2: Peripheral venous thrombectomy imaging before and after intervention.**

Supplemental Figure 2**:** Representative peripheral venographic images obtained before (left panel) and after (right panel) thrombectomy in a patient with lower-extremity venous thrombosis.

**Supplemental Figure 3: PE-related thrombectomy imaging before and after intervention**

Supplemental Figure 3**:** Representative pulmonary angiographic images obtained before and after thrombectomy in a patient analyzed in this study that presented with concomitant pulmonary embolism.

**Supplemental Figure 4: CLEANER XT™ Rotational Thrombectomy System Overview (Argon Medical Devices)**

CLEANER™ Rotational Thrombectomy System | Argon Medical

Supplemental Figure 4**:** Detailed view of the CLEANER XT™ system, demonstrating the sinusoidal wire mechanism for thrombus maceration prior to aspiration with the CLEANER Vac™. (Image courtesy of Argon Medical Devices.)
