# Supplementary material for: ARC-VAC Study (Analysis of real-world multicenter experience with the large-bore CLEANER-Vac Thrombectomy System): An analysis of 200 patients
Source: J Vasc Surg Venous Lymphat Disord. 2026 Sep 28;15(1):102634. doi: 10.1016/j.jvsv.2026.102634 (PMC13720463; doi:10.1016/j.jvsv.2026.102634)
Supplement: Supplementary file 2 [file mmc2.docx]

**Supplemental Table I: Baseline Demographic and Clinical Characteristics (N=200)**

| **Characteristic** | **Value** |
| --- | --- |
| **Age, years** |  |
| Mean ± SD | 64.5 ± 13.8 |
| Range | 25–90 |
| **Gender, n (%)** |  |
| Female | 104 (52.0) |
| Male | 96 (48.0) |
| **Thrombus Chronicity, n (%)** |  |
| Acute (<14 days) | 130 (65.0) |
| Subacute (14–28 days) | 70 (35.0) |
| **Thrombus Location, n (%)** |  |
| Lower extremity | 140 (70.0) |
| Iliofemoral | 90 (45.0) |
| Femoropopliteal | 50 (25.0) |
| Upper extremity | 30 (15.0) |
| Pulmonary | 15 (7.5) |
| Other (e.g., portal, splenic) | 15 (7.5) |
| **Comorbidities, n (%)** |  |
| Malignancy | 48 (24.0) |
| Recent surgery/trauma | 40 (20.0) |
| Immobility | 36 (18.0) |
| Hypercoagulable state | 32 (16.0) |
| Prior VTE | 24 (12.0) |
| **Concomitant Active PE, n (%)** | 15 (7.5) |
| Intermediate high-risk PE | 15 (7.5) |

Supplemental Table I. Baseline characteristics of the 200-patient cohort treated with the CLEANER Vac™ Thrombectomy System. VTE = venous thromboembolism; PE = pulmonary embolism. Percentages may not sum to 100% due to rounding.

**Supplemental Table II: Procedural Characteristics (N=200)**

| **Procedural Variable** | **Value** |
| --- | --- |
| **Venous Segments Intervened, mean ± SD (range)** | 2.2 ± 1.0 (1–5) |
| **Vascular Access Site, n (%)** |  |
| Jugular | 60 (30.0) |
| Femoral | 70 (35.0) |
| Alternative (primarily popliteal) | 70 (35.0) |
| **Adjunctive Interventions, %** |  |
| Rotational morcellation (CLEANER 15™) | 30.0 |
| Balloon Venoplasty | 38.0 |
| Iliac venoplasty | 50 (25.0; 65.8% of venoplasty) |
| Common femoral/lower common femoral venoplasty | 21 (10.5; 27.6% of venoplasty) |
| Subclavian venoplasty | 5 (2.5; 6.6% of venoplasty) |
| Stenting | 15.0 |
| Left iliac stenting | 26 (13.0; 86.7% of stented) |
| Right iliac stenting | 4 (2.0; 13.3% of stented) |
| Catheter-directed thrombolysis (abbreviated) | 5.0 |
| **Procedural Metrics** |  |
| Mean device utilization time, min ± SD | 8.0 ± 3.2 |
| Mean device passes, n ± SD | 2.4 ± 1.1 |
| Mean total procedural duration, min ± SD | 68.0 ± 22.0 |
| Single-session completion, % | 94.0 |

Supplemental Table II. Procedural details for the CLEANER Vac™ Thrombectomy System in 200 patients. Adjunctive interventions were used at operator discretion; some patients received multiple adjuncts. Venoplasty and stenting categories are not mutually exclusive because stent reconstruction may include balloon venoplasty for lesion preparation or optimization of stent expansion.

**Supplemental Table III: Efficacy Outcomes (N=200)**

| **Outcome Measure** | **Value** |
| --- | --- |
| **Clot Burden, mean ± SD (%)** |  |
| Pre-procedure | 88.0 ± 11.5 |
| Post-procedure | 10.5 ± 10.8 |
| **Thrombus Clearance, mean ± SD (%)** | 88.0 ± 13.2 |
| **Thrombus Clearance Thresholds, n (%)** |  |
| ≥90% resolution | 152 (76.0) |
| ≥75% resolution | 184 (92.0) |
| **Segment-Specific Clearance, mean ± SD (%)** |  |
| Iliac | 89.5 ± 12.0 |
| Femoral | 87.2 ± 13.5 |
| Popliteal | 88.8 ± 12.8 |
| **Clearance by Thrombus Acuity, mean ± SD (%)** |  |
| Acute | 89.2 ± 12.5 |
| Subacute | 86.5 ± 13.8 |

Supplemental Table III. Primary efficacy endpoints. Clot burden was quantified per segment via venography and averaged per patient.

**Supplemental Table IV: Safety Outcomes (N=200)**

| **Safety Parameter** | **Value** |
| --- | --- |
| **Estimated Blood Loss (EBL), mL** |  |
| Overall mean ± SD (range) | 320 ± 140 (50–750) |
| By Operator Experience* |  |
| >10 procedures (n=120 cases) | 185 ± 55 (150–220) |
| ≤10 procedures (n=80 cases) | 380 ± 160 |
| **Complications, n (%)** | 5 (2.4) |
| Minor access-site hematomas | 5 (2.4) |
| **Device-Related Serious Adverse Events** | 0 (0.0) |
| **Mortality (unrelated)** | 1 (0.5) |

Supplemental Table IV. Safety profile. *P<0.01 for experienced vs. novice operators (unpaired t-test). No transfusions required.
